# Circadian dynamics in measures of cortical excitation and inhibition balance

**DOI:** 10.1038/srep33661

**Published:** 2016-09-21

**Authors:** Sarah L. Chellappa, Giulia Gaggioni, Julien Q. M. Ly, Soterios Papachilleos, Chloé Borsu, Alexandre Brzozowski, Mario Rosanova, Simone Sarasso, André Luxen, Benita Middleton, Simon N. Archer, Derk-Jan Dijk, Marcello Massimini, Pierre Maquet, Christophe Phillips, Rosalyn J. Moran, Gilles Vandewalle

**Affiliations:** 1 GIGA-Research, Cyclotron Research Center-In Vivo Imaging Unit, 8 allée du Six Août, Batiment B30, University of Liège, 4000 Liège, Belgium; 2 Walloon excellence in life sciences and biotechnology (WELBIO), Belgium; 3 Department of Neurology, Domaine Universitaire du Sart Tilman, Bâtiment B35, CHU de Liège, 4000 Liège, Belgium; 4 Department of Biomedical and Clinical Sciences “E.Sacco”, Università degli Studi di Milano, via G. B. Grassi 74, 20157 Milano, Italy; 5 Fondazione Europea di Ricerca Biomedica, Ferb Onlus, Milan, Italy; 6 Surrey Sleep Research Centre, Faculty of Health and Medical Sciences, University of Surrey, GU2 7XP Guildford, United Kingdom; 7 Virginia Tech Carilion Research Institute & Bradley Department of Electrical and Computer Engineering, Virginia Tech, 2 Riverside Circle, VA 24016 Roanoke, USA

## Abstract

Several neuropsychiatric and neurological disorders have recently been characterized as dysfunctions arising from a ‘final common pathway’ of imbalanced excitation to inhibition within cortical networks. How the regulation of a cortical E/I ratio is affected by sleep and the circadian rhythm however, remains to be established. Here we addressed this issue through the analyses of TMS-evoked responses recorded over a 29 h sleep deprivation protocol conducted in young and healthy volunteers. Spectral analyses of TMS-evoked responses in frontal cortex revealed non-linear changes in gamma band evoked oscillations, compatible with an influence of circadian timing on inhibitory interneuron activity. *In silico* inferences of cell-to-cell excitatory and inhibitory connectivity and GABA/Glutamate receptor time constant based on neural mass modeling within the Dynamic causal modeling framework, further suggested excitation/inhibition balance was under a strong circadian influence. These results indicate that circadian changes in EEG spectral properties, in measure of excitatory/inhibitory connectivity and in GABA/glutamate receptor function could support the maintenance of cognitive performance during a normal waking day, but also during overnight wakefulness. More generally, these findings demonstrate a slow daily regulation of cortical excitation/inhibition balance, which depends on circadian-timing and prior sleep-wake history.

Neuronal function sustaining human cognition depends on the balanced and recurrent activity of excitatory and inhibitory circuits^1^. Interconnected networks of excitatory pyramidal neurons, inhibitory interneurons and excitatory stellate cells orchestrate cortical oscillations^2^, thus shaping cortical and behavioral responses^3^. This orchestration is homeostatically regulated at the millisecond-to-second time-scale to allow for neuron depolarization and repolarization and to maintain synaptic and neuronal activity within an appropriate range^4^,^5^. However, brain function is also regulated on a much slower time-scale. Sleep homeostasis, prior sleep-wake history, and circadian processes interact and regulate cognitive brain function across the course of a day^6^,^7^. This interaction maintains stable cognitive performance during a normal ~16 h waking day, despite the changes in neuronal structure and the intra/extracellular milieu^8^ associated with wakefulness, because of a circadian drive for wakefulness which progressively opposes sleep need accumulation, up to the evening hours. At night, the circadian system favors sleep such that, if wakefulness is extended overnight, the deleterious effects of prolonged wakefulness are emphasized by the circadian drive for sleep^9^. Conversely, in the morning following a night without sleep, performance stabilizes because the circadian system switches back towards wakefulness promotion^9^. Despite considerable advances in the understanding of the cerebral ground of this slow daily regulation of brain function using scalp EEG and neuroimaging^10^, the impact of sleep homeostasis and circadian processes on the dynamics of excitatory and inhibitory circuits are not established.

It is commonly accepted that an increase in excitation is invariably accompanied by an increase in inhibition and vice-versa^3^. However, recent data acquired in Drosophila, Zebrafish and rodents posit that sleep homeostasis and circadian processes influence synaptic efficacy and morphology^8^,^11^,^12^,^13^,^14^,^15^. Likewise, human studies reported that cortical excitability, i.e. amplitude and slope of scalp EEG responses to stimulations, is not stable and depends on both sleep homeostasis and the circadian system^16^,^17^. In addition, rodent and human data, respectively, showed changes in GABAergic function during sleep^18^ and in glutamatergic receptor density following sleep deprivation^19^. Human data also suggest time-of-day changes in corticospinal excitability, independent of sleep, through GABA inhibitory networks^20^. It is therefore likely that the balance between excitation and inhibition drives changes during the 24 h day.

Addressing directly this issue in humans remains a challenge, however. Most of what is currently known about the *in vivo* daily dynamics of neuronal ensemble is based on animal data^8^,^11^,^21^,^22^. In humans, pharmacological manipulations^23^ and intracerebral electrode implantation in pre-surgical epilepsy^24^,^25^ stand as the main sources of information on this aspect of brain function. Coupled TMS pulses (a few ms apart) have also been used to isolate inhibitory or excitatory activity based on TMS induced motor response^26^. It remains nevertheless difficult to isolate simultaneously excitation and inhibition drives from different neuronal subpopulation *in vivo*, even using invasive approaches, such as intracranial EEG recordings in patients^24^,^25^,^27^. Computational models stand, therefore, as one of the useful non-invasive approaches to access excitation/inhibition.

Here, our aim is to assess the dynamics in human cortical excitation/inhibition balance over the 24 h day. We report results of an original analysis extension of previous data which consisted in neuropsychological and neurophysiological assessments during a 29 h sleep deprivation protocol in young and healthy individuals^17^. We first use spectral power of TMS-evoked EEG responses as an index excitation/inhibition function. We then apply a physiologically validated *in silico* model to the TMS-evoked EEG responses to infer a more detailed mesoscopic description of excitation/inhibition within a cortical column. We hypothesize that circadian phase would influence excitation and inhibition balance and that this influence would be most apparent through a non-linear, ~24 h sine-wave oscillatory patterns in excitation/inhibition parameters. We further postulated that this mesoscopic non-linear neuronal network dynamics would be related to changes in cortical excitability and behavioral measures acquired during the sleep deprivation protocol.

## Results

**Figure 1:**
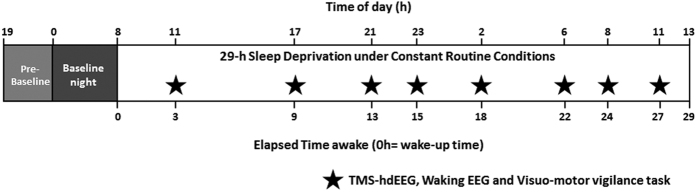
Experimental protocol. Twenty-two healthy young men (22.8 ± 2.6 y.o.) underwent 8 TMS/EEG sessions during 29-h sleep deprivation to cover the ~24 h circadian cycle under constant routine conditions. EEG recordings of TMS evoked responses were distributed with a higher frequency around the so-called evening “wake maintenance zone”, which occurs before melatonin onset and corresponds to the periods when the circadian system maximally favors wakefulness. Higher frequency also occurred in the morning “sleep promoting zone”, when the circadian system maximally promotes sleep, before it favors wakefulness again. During TMS/EEG sessions, participants performed a visuo-motor vigilance task. TMS/EEG sessions were immediately preceded by 2-min recordings of spontaneous quiet waking EEG.

Following an 8-h nocturnal baseline sleep, 22 healthy young men (22 ±  2.6 y.o.; Tables 1 and 2), underwent 8 TMS/EEG sessions during approximately 29-h of sustained wakefulness. The study paradigm used controlled behavioral and environmental conditions (constant routine protocol) to minimize external and internal factors that may potentially mask circadian rhythmicity^28^ (Fig. 1). EEG recordings of TMS evoked responses were timely distributed with a higher frequency around the periods with higher non-linearity in the circadian signal, i.e. when it is expected to switch from wakefulness to sleep promotion and vice-versa^29^. The frontal cortex was chosen as stimulation target due to its exquisite sensitivity to increased sleep pressure^30^. [Table t2]

**Figure 2:**
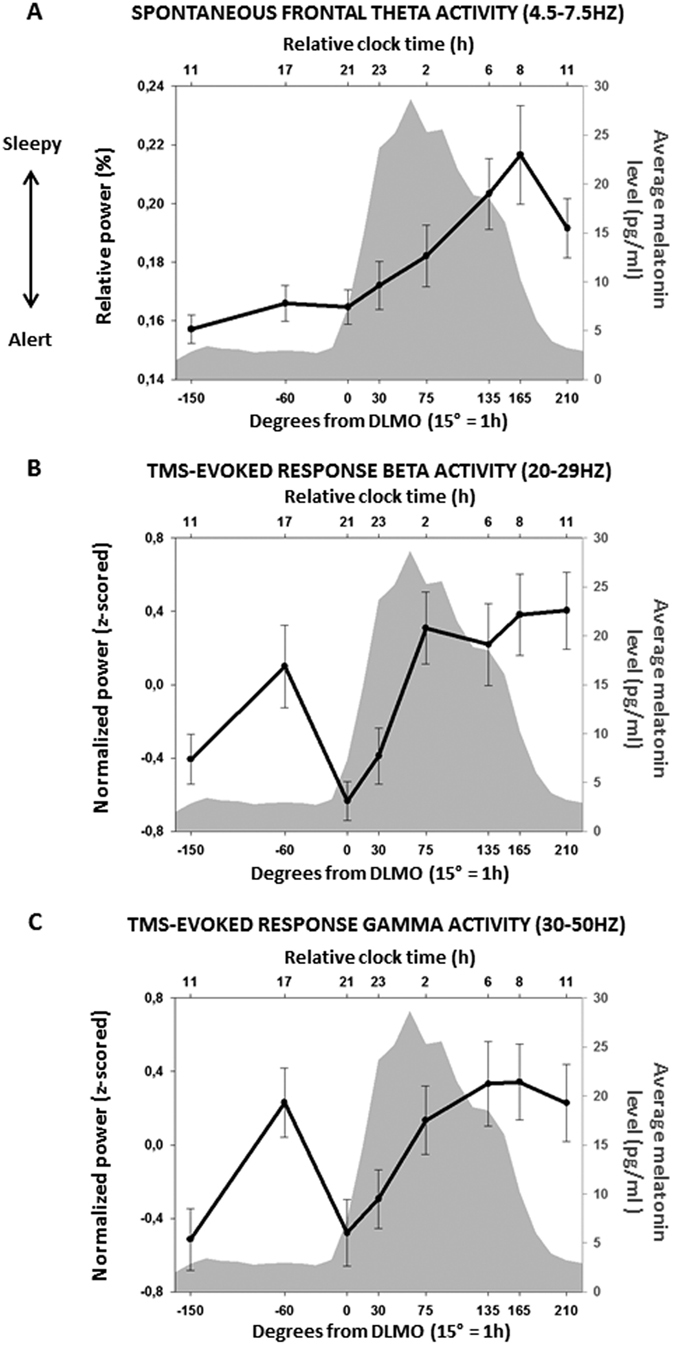
Dynamics of macroscopic, spontaneous EEG theta activity and TMS evoked EEG beta and gamma activity over time. (**A**) Spontaneous waking EEG Theta activity (4.5–7.5 Hz; normalized to the sum of EEG activity from 0.75–20 Hz) significantly varied over time as previously reported^17^. It remained stable up to the circadian wake-maintenance zone and increased during the circadian sleep-promoting zone. (**B**) TMS-evoked Beta EEG activity (z-scored sum of 20–29 Hz) at the closest electrode from hotspot (whose location was provided by the neuronavigation system) significantly varied across time, with nadir around the wake-maintenance zone and peak during the sleep-promoting zone. (**C**) TMS-evoked Gamma EEG activity (z-scored sum of 30–50 Hz) at the closest electrode from hotspot significantly varied across time, with nadir around the wake-maintenance zone and peak during the sleep-promoting zone. N = 22, on all figures and data are realigned according to individual melatonin secretion onset (phase 0°). Bottom horizontal axis corresponds to time in degrees (15° = 1 h) relative to melatonin secretion onset. Top horizontal axis correspond to the corresponding relative clock time (in hours) for an individual habitually sleeping at 11PM and waking up at 7AM. Left vertical axis corresponds to mean ± Standard deviation (SD). Gray shade corresponds to averaged melatonin values (pg/ml - right vertical axis).

We first focus on the 2-minute EEG recording of eye-opened spontaneous waking activity to assess the evolution of theta power, a gold standard objective EEG measure of sleepiness/alertness level^9^,^31^. As expected^9^,^31^, theta power (4.5–7.5 Hz) significantly varies (F_7,103_ = 3.73, *p* = 0.001) following a typical non-linear variation across the protocol (Fig. 2A), reflecting a dual influence from time awake, i.e. sleep homeostasis, and circadian phase on alertness level (see Supplementary Fig. S1 for other frequency bands). Theta power is stable up to the evening hours, corresponding to the evening “wake-maintenance zone”, i.e. when the circadian system maximally favors wakefulness^29^, at melatonin secretion onset (circadian phase 0°). Theta power then increases during the biological night, i.e. when the circadian system favors sleep^29^, prior to decreasing again in the morning hours.

### Changes in EEG synchrony speak to modifications in excitation/inhibition balance during prolonged wakefulness

The literature posits that different cortical areas oscillate in their intrinsic EEG frequency modes in response to TMS stimulations^32^. Accordingly, the frontal cortex, which was the cortical area stimulated in our protocol, oscillates in the fast beta band (20–29 Hz) when pertubated by TMS pulses. Similarly to theta power, data show that beta power significantly varies with wakefulness extension (F_7,130_ = 3.64, *p* < 0.001) following a non-linear temporal profile (Fig. 2B), with nadir evident around the evening “wake-maintenance zone” (session 0° is significantly different from the mean; p_corrected_ = 0.01). This initial decrease was followed by a sharp increase during the biological night (sessions 135° and 165° tend to be significantly different from the mean; p_corrected_ = 0.08). This finding reflects that extended wakefulness is associated with changes in a fundamental mechanism shaping TMS-induced responses.

We next assessed whether EEG power in the gamma band of the TMS-evoked response (30–50 Hz), which has been related to GABA activity of inhibitory interneurons^23^, changed across the 29 h of prolonged wakefulness. Analyses reveal that gamma power undergoes significant non-linear changes with time awake (F_7,131_ = 4.62, *p* < 0.001), similar to the non-linear profile observed in the beta band (Fig. 2C). A marked decrease in gamma power is observed around the evening wake-maintenance zone (session 0° is significantly different from the mean; p_corrected_ < 0.001) followed by a steep increase during the biological night (session 165° is significantly different from the mean; p_corrected_ = 0.05).

### Model-based assessments of excitation and inhibition in a cortical column follow a circadian profile

**Figure 3:**
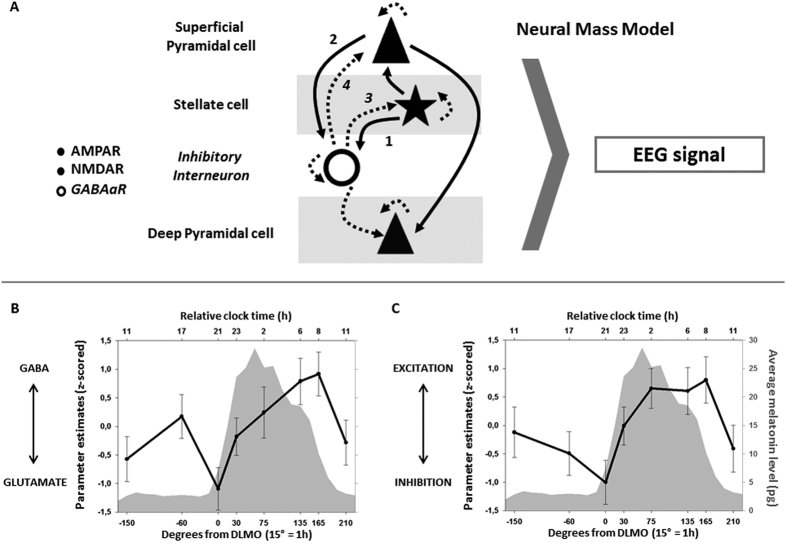
Dynamics of GABA/Glutamate receptor time constant and cell-to-cell excitation/inhibition connectivity balances during normal waking and sleep deprivation. (**A)** Neural Mass Modeling in Dynamic Causal Modeling (DCM) decomposes a cortical area into 4 neuronal subpopulations: superficial and deep pyramidal cells, spiny stellate cells and inhibitory interneurons. Each subpopulation projects to the other subpopulations via excitatory (**solid lines**) and inhibitory (**dashed lines**) connections, and have inhibitory feedback-loops controlling neuronal gain. Furthermore, DCM include 3 common synaptic ionotropic receptors time constants (AMPA, NMDA, GABAa receptor). To derive putative markers of excitation/inhibition balance, we derived two key indices. The first index comprised the time constants of GABAaR, and glutamatergic AMPAR and NMDAR. The second index used cell-to-cell population connectivity parameters to estimate connectivity strength and balance between stellate cells, deep pyramidal cells and inhibitory interneurons. (**B)** GABA/Glutamate receptor time constant balance (z-scored parameters) varied significantly with time, with more glutamatergic drive around the circadian wake-maintenance zone and more GABAergic drive during the biological night. (**C)** Excitation/inhibition cell-to-cell connectivity parameter balance (z-scored parameters) varied significantly across time, with relatively more inhibition around the circadian wake-maintenance zone and relatively more excitation during the biological night.

We then applied an *in silico* approach to infer excitation/inhibition balance using Dynamic Causal Modelling (DCM) for event related potentials (Fig. 3A)^33^. DCM has been extensively used in animal and human experiments, including in conjunction with pharmacological manipulations or invasive intracortical recordings, and has allowed, for instance, comprehension of how brain dynamics underpin cognition^34^ and different states of consciousness^35^. In essence, DCM^33^ provides a framework for effective connectivity analyses among neuronal subpopulations that underlie invasive and non-invasive (EEG) electrophysiological responses^35^,^36^,^37^. In broad terms, neural mass models assume that neuronal states are comprised of numerous features (membrane potentials, ionic-conductance, pre- and post-synaptic responses, and so forth) which are inferred within a given cortical area comprising 4 subpopulations of neurons (deep and superficial pyramidal cells, excitatory stellate cells and inhibitory interneurons). These subpopulations have excitatory and inhibitory connections among each other and also exhibit self-inhibition controlling neuronal gain. Furthermore, the model includes the time constant of 3 of the most common synaptic ionotropic receptors: GABA_A_ receptors (GABAaR), α-amino-3-hydroxy-5-methyl-4-isoxazolepropionic acid receptors (AMPAR) and N-methyl-D-aspartate (NMDAR) receptors^36^. The model variables are hidden, i.e. they are not directly measured, but are inferred from electrophysiological time-series using forward models for evoked EEG responses and fits of these models using a Bayesian inversion scheme (i.e. EEG source reconstruction)^33^. Here, we used DCM to compute two indices of excitation/inhibition balance based on 5–50 ms post-TMS evoked EEG responses.

The first index comprised the relative time constants of inhibitory GABAaR and excitatory glutamatergic AMPAR and NMDAR [GABAaR *minus* AMPAR+NMDA]. Both GABAergic and glutamatergic receptors are indeed likely affected by sleep-wake history and time of day^18^,^19^. Analysis revealed that the balance between GABA/Glutamate receptor time constant, as assessed using DCM, significantly varies over time (F_7,130_ = 2.8, *p* = 0.01; Fig. 3B; for individual data see Fig. S2A). In addition, its 24 h temporal dynamic is non-linear. A local relative increase in glutamatergic over GABAergic receptor time constant occurs around the evening wake-maintenance zone (session 0° is significantly different from the mean; p_corrected_ = 0.005). Conversely, GABAergic receptor time constant undergoes a large increase relative to glutamatergic receptor during the biological night (sessions 135°/165° are significantly different from the mean; p_corrected_ ≤ 0.04). GABA/glutamate receptor time constant value then returns towards mean GABA vs. glutamatergic parameter levels.

The recurrent coordination of excitation and inhibition within neural networks is subtended by the connectivity between excitatory pyramidal cells and stellate cells and inhibitory interneurons^2^. Hence, we computed a second index which used cell-to-cell population connectivity parameters to estimate connectivity strength and balance between stellate cells, deep pyramidal cells and inhibitory interneurons., i.e. the major neuronal subpopulations underscoring excitation/inhibition mechanisms within a cortical column^2^ [sum of excitatory connectivity from stellate and deep pyramidal cells to inhibitory interneurons *minus* sum of inhibitory connectivity from inhibitory interneurons to stellate and superficial pyramidal cells]. Similarly to the receptor time constant index, analysis show that neuronal cell-to-cell excitation/inhibition balance significantly varies with non-linear dynamics during the 29 h sleep deprivation protocol (F_7,130_ = 3.1, *p* = 0.006; Fig. 3C; for individual data see Fig. S2B). Inhibitory connectivity first increases relative to excitatory connectivity up to the evening wake-maintenance zone (session 0° is significantly different from the mean; p_corrected_ = 0.01). During the biological night, however, excitatory connectivity increases relative to inhibitory connectivity up to the early morning sleep promoting zone (session 165° is significantly different from the mean; p_corrected_ = 0.04).

Therefore, intriguingly, it is as if both indices go in opposite directions, seemingly “counteracting” each other. Interestingly also, both neuronal indices seemed to “recover” in the morning following sleep deprivation, when the circadian system switches back to favoring wakefulness: values of both morning sessions (i.e. 24 h apart, at the same circadian phase but under low and high sleep pressure) are indeed not statistically different from the mean (*p* > 0.14). Thus, the dynamics of neuronal excitation/inhibition balance within a cortical region appears to be under a strong circadian influence.

### Circadian dynamics of excitation/inhibition balances is associated with scalp EEG synchrony, cortical excitability and behavior

**Figure 4:**
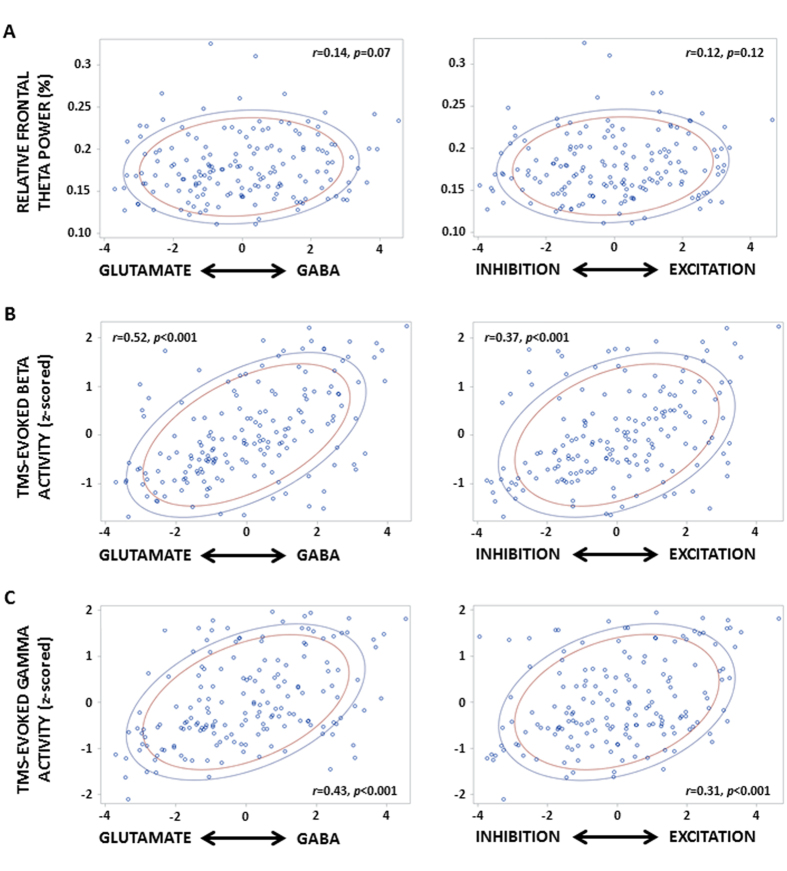
Dynamics of GABA/Glutamate receptor time constant and of cell-to-cell excitation/inhibition balances are related to macroscopic neurophysiological changes. Correlations between GABA/Glutamate receptor time constant and cell-to-cell excitation/inhibition connectivity balances and relative frontal theta (4.5–7.5 Hz) power of the spontaneous EEG recording (**A**), and beta (20–29 Hz) (**B**) and gamma (**C**) power of the TMS evoked EEG responses. In all correlations, n = 22, and blue and red lines correspond, respectively, to 80% and 70% of data (prediction ellipses); r and p values are displayed on each panel.

Next, we investigated whether the non-linearity in neuronal balance could potentially impinge on system-level changes. We first considered theta power of the spontaneous EEG recordings (Fig. 4A), as an objective marker of alertness level^9^. Correlation tends to be significant with GABA/glutamate receptor time constant (Spearman correlations; *r* = 0.14, p = 0.07) but not with excitatory/inhibitory cell-to-cell connectivity balances (Spearman correlations; *r* = 0.12, p = 0.12). No significant correlations are found with power in other frequency bands of the spontaneous waking EEG recordings (Fig. S1). In contrast, both DCM indices are significantly associated with TMS-evoked beta EEG activity level, the intrinsic oscillatory mode of the frontal cortex^32^ (Spearman correlations; with GABA/glutamate receptor density balance: *r* = 0.52, *p* < 0.001; with cell-to-cell excitation/inhibition balance *r* = 0.37, *p* < 0.001; Fig. 4B).

Further correlation analyses indicate that a relative increase in GABAa receptor over glutamatergic receptor time constant, as inferred from our *in silico* approach, is correlated to more GABA activity of inhibitory interneurons, as indexed through the EEG power in the gamma band^23^ (Spearman correlations; *r* = 0.43, *p* < 0.001; Fig. 4C). In contrast, more inhibitory connectivity within a cortical column, as indexed through DCM, is associated with less GABA activity of interneurons, based on power of the gamma band (Spearman correlations; *r* = 0.31, *p* < 0.001).

**Figure 5:**
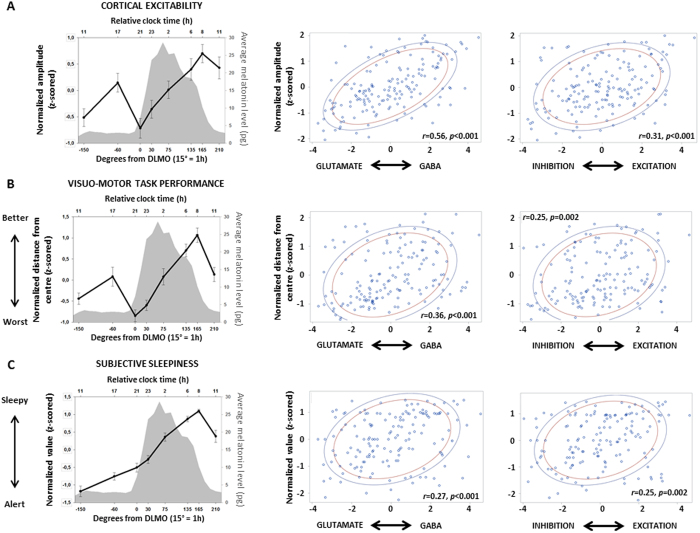
Dynamics of Neuronal excitation/inhibition and GABA/Glutamate receptor balance are related to cortical excitability and behavior. *Left panels:* Cortical excitability, as indexed by the amplitude of the early (0–30 ms) TMS-evoked EEG response (z-scored; slope not shown) (**A**), performance to the visuo-motor vigilance task (z-scored mean distance to center of the screen) (**B**) and subjective sleepiness scores (z-scored) (**C**) significantly varied with time awake as reported in ref. 17. GABA/Glutamate receptor time constant (*middle panels*) and cell-to-cell excitation/inhibition connectivity (*right panels*) balances significantly correlated with cortical excitability (**A**), performance to the visuo-motor vigilance task (**B**) and subjective sleepiness scores (**C**). In all correlations, n = 22, and blue and red lines correspond, respectively, to 80% and 70% of data (prediction ellipses); r and p values are displayed on each panel.

Likewise, GABA/glutamate receptor time constant balance strongly and significantly correlates with both the amplitude and slope of the TMS evoked responses (Spearman correlations; amplitude: *r* = 0.56, *p* < *0.001*; slope: *r* = 0.52, p < 0.001; Fig. 5A, only amplitude is displayed), a proxy for cortical excitability^17^. Excitatory/inhibitory cell-to-cell connectivity balances is also significantly correlated with both indices of cortical excitability (Spearman correlations; amplitude and slope: *r* = 0.31, *p* < 0.001; Fig. 5A).

The ultimate role of neuronal activity is to coordinate overt behavior^3^. We therefore pursued by focusing on performance to a task acquired simultaneously with each TMS/EEG recordings. This visuo-motor vigilance task consists of maintaining a constantly moving red dot in the center of a black screen^16^,^17^. Average distance kept to the center significantly changes across time in a non-linear manner (F_7,122_ = 13.78; p < .0001), thus mirroring putative sleep and circadian mechanisms (Fig. 5B). Both GABA/glutamate receptor time constant (Spearman correlations; *r* = 0.36, *p* < 0.001) and cell-to-cell excitation/inhibition connectivity (Spearman correlations; *r* = 0.25, *p* = 0.002) balances are significantly associated with performance to the task (Fig. 5B). Excitation/inhibition balance indices are also related to another aspect of behavior as a significant correlation is detected between both DCM indices and subjective ratings of sleepiness obtained right after each TMS-EEG assessments (Spearman correlations; with GABA/glutamate receptor time constant balance: *r* = 0.27, *p* < 0.001; with cell-to-cell excitation/inhibition balance *r* = 0.25, *p* < 0.002; Fig. 5C).

Finally, we probed whether all these correlations would vary as a function of circadian phase by performing analyses of covariance on the same combination of variables as those used for correlations, but including circadian phase as a factor. Analyses of covariance confirm all previous significant correlations, as they lead to significant covariance with DCM parameters irrespective of circadian phase (*p* < 0.002) (TMS-evoked beta and gamma power, cortical excitability, visuo-motor task performance, subjective sleepiness). Ancovas also indicate significant associations between power in the theta frequency band of the spontaneous waking EEG and both DCM indices (*p *≤ 0.05). Associations with other frequency bands of the spontaneous waking EEG remained non-significant (p > 0.15). Importantly, the relationship between our markers for neuronal excitation/inhibition balance and measures of system-level physiology and behavior are similar across all circadian phases for all parameters considered (interaction of with circadian phase, *p* > 0.15).

## Discussion

The findings of the present study provide a spectral and model-based decomposition of circadian regulation of neuronal ensemble activity in the human brain *in vivo.* Both spectral and model-based indices reveal a slow, daily regulation of excitation/inhibition balance within a cortical column, which operates at a different time-scale than established fast homeostatic regulation^3^.

We first show that EEG power of TMS-evoked responses in the beta and gamma bands showed marked slow temporal variations most likely stemming from circadian rhythmicity and sleep need variations. These might be seen as proxies for activity intrinsic to the frontal cortex^32^ and GABA activity of inhibitory interneurons^23^, respectively. These macroscopic events must be underscored at the mesoscopic level, through changes in neuronal dynamics. We therefore adopted a computational approach to infer putative details of excitatory and inhibitory processes within a cortical column. We provide evidence that both the index of GABA-glutamate main receptor time constant and the index of cell-to-cell excitatory-inhibitory connectivity, as inferred from a realistic data-driven computational model^33^,^38^, appear to be under strong circadian influence. Future experiments will investigate inter-individual variability in the variations in both indices. The data also show that these mesoscopic circadian fluctuations seem to translate to the previously reported change in cortical excitability^17^ and to the variations in the beta and gamma EEG frequency bands. These nonlinear changes together could constitute the mechanistic bases of the well characterized non-linear changes in neurobehavioral performance and cognition during prolonged wakefulness^6^,^9^.

According to our data, the first 16 h of a normal waking day are accompanied by a relative increase in glutamatergic vs. GABAergic receptor time constant. This finding is reminiscent of the rise in glutamate receptor subtypes previously observed in the late afternoon, following 33 h without sleep in humans^19^ and could also be linked to the progressive increase in extracellular glutamate levels reported in rodents during normal waking hours^22^. In concert to this relative increase in excitatory receptor density, inhibitory cell-to-cell connectivity drive is enhanced relative to excitatory connectivity drive. This increase of inhibitory interneuron connectivity could therefore stand as a novel circadian means through which changes in synaptic receptor composition and in extracellular milieu are faced to stabilize neuronal activity during normal-duration wakefulness.

Sleep deprivation represents a challenging circumstance whereby circadian control desynchronizes from behavioral states: at a time when we are biologically tuned for sleep, we are awake. In this setting, neuronal events that typically occur during sleep might happen during wakefulness. Rodent data posit that the expression of genes encoding for GABAa receptor levels increases during sleep and this would in turn facilitate synaptic downscaling^18^. Furthermore, mRNA levels for selected subunits of GABAa receptors within the posterior hypothalamus of rats were reported to be higher at the end of the active period or following sleep deprivation, when the need for sleep is high^39^. Here, we observe a similar night-time relative increase in GABAa receptor time constant but in the cortex and in the absence of sleep. We postulate therefore that an overnight GABAa receptor time constant increase arise from an increase in its gene expression and could be one facet of the circadian signal favoring sleep and may not be exclusively attributable to sleep itself.

Our data further suggest that during “abnormal” overnight wakefulness, this putative circadian change in inhibitory receptor time constant is countered by a relative increase in excitation vs. inhibition in terms of cell-to-cell connectivity within a cortical column. Under the unfavorable circumstance of sleep deprivation during the biological night, this net excitatory cell-to-cell increase could compensate and allow cognition to unfold in frontal regions. This net excitatory drive increase would not allow for optimal conditions such that performance decreases^17^. We cannot test, however, for causal influences within the model itself. Our data indicate nevertheless a relationship between slow changes in cortical excitation and inhibition balance over the course of wakefulness, and the slow variations in cognitive performance as well as in systems-level frontal synchronization. Importantly, however, our computational approach does not explicitly include important neuromodulators which are key drivers of connectivity change or time constant adjustment, such as norepinephrine, serotonin, acetylcholine or dopamine, and are central to sleep-wake regulation^40^.

Subjective sleepiness was significantly correlated with change in DCM indices of excitation/inhibition balance, as well as oscillations in the theta band of spontaneous EEG activity, an established marker of alertness/sleepiness levels^31^, when using Ancovas taking circadian phase into account (tendency or non-significant relationship with simple correlations). This suggests that the dynamics in objective and subjective measures of sleepiness are potentially related to changes in excitation/inhibition balance among cortical neurons, but this will deserve further investigation.

GABA agonists are among the most common prescribed neuroactive compounds because they constitute the vast majority of sleeping pills^18^,^41^. In addition, several under-development compounds for cognitive enhancement are actually targeting GABA or glutamate receptors^42^. Both the latter clinical trials and benzodiazepine prescription could potentially be improved by taking into account slow 24 h change in excitation/inhibition balance. Furthermore, the slow 24 h changes in cortical excitation/inhibition balance may contribute to the well-documented time-of-day variation in seizure occurrence in certain forms of epilepsy^43^.

Overall, our findings support that sleep is not the exclusive factor for a return of neuronal parameters to baseline values in the morning. In fact, both aspects of excitation/inhibition balance we focused on through our modeling approach do not appear to need sleep at all to recover baseline levels. Therefore, contrary to performance, excitation/inhibition balance within a cortical column could be under a prominent circadian influence. Thus, the dual influence of sleep-wake history and circadian phase on brain function could impact differently distinct aspects of cortical function.

## Methods

The protocol and part of the analyses are already described in ref. 17.

### Participants

Participants gave written informed consent and received a financial compensation. The study was approved by of the University of Liège Ethics Committee and followed all the Belgian and European guidelines and regulations with respect to human scientific research. Twenty-four healthy young men (18–30 years old) were enrolled. Exclusion criteria included: (1) BMI ≤18 and ≥25; (2) psychiatric history, severe head trauma, sleep disorders; (3) addiction, chronic medication; (4) smoking, excessive alcohol consumption (>14 doses per week) or caffeine (>3 cups/day); (5) night shift workers during the last year; (6) transmeridian travel (>1 time zone) <2 months; (7) anxiety and/or depression. One participant was excluded from analyses due to melatonin phase-delay >6 h compared with the average of the sample, and one due to artifacts in EEG recordings throughout the protocol. Thus, data presented here include 22 participants (Tables 1 and 2 for detailed participant’s characteristics).

### Study protocol

EEG cortical responses evoked by a TMS pulse directly probe cortical, or cortico-thalamic, response bypassing any potential sensory bias^32^, in contrast to TMS-evoked motor response assessment, which depends on transmission through corticospinal and neuromuscular pathways.

Participants first completed a “pretest” TMS/EEG session to determine the optimal TMS parameters providing artefact-free EEG recordings. The left or right supplementary motor area (SMA) was set as stimulation target for right or left-handed, respectively. This brain area was identical to^30^ and was chosen for the following reasons: (1) similar to the entire frontal lobe, the SMA is exquisitely sensitive to sleep pressure, including at the neuronal level, as indicated in a previous EEG-TMS experiment^30^; (2) it plays a key role in cognitive performance, and is heavily connected to the prefrontal cortex^44^; (3) its stimulation does not trigger muscle activation, sources of EEG signal contamination (this was verified online for each pretest and experimental sessions).

Participants then underwent one laboratory polysomnography night to exclude any sleep disorders. Afterwards, they kept a regular sleep-wake schedule of 8 h sleep duration (+/−15 min) 1-week prior to the study. Compliance was verified using wrist actigraphy (Actiwatch, Cambridge Neuroscience, UK) and sleep diaries (Table 1). Schedule was individual set based on habitual sleep and wake times and all aspects of the in-laboratory experiment were timed according to individual habitual sleep and wake times.

For the experimental setup *per se*, participants arrived at the laboratory 6 h prior to sleep time and were maintained in dim-light (<5 lux) thereafter. They were trained on the behavioral test battery before sleeping (in darkness) at their habitual bedtimes under EEG (Fig. 1A). They then remained awake for 29 h during which they continuously wore a TMS-compatible 60 electrode EEG cap and were under constant routine conditions to minimize external and internal factors potentially masking circadian rhythmicity^28^ [constant semi-recumbent position, temperature, dim light (<5 lux), isocaloric food intake every 2 h, sound proof rooms]. Quiet spontaneous waking EEG (2 min with blink suppression) and TMS evoked EEG potentials were recorded (>250 trials) 8 times during sleep deprivation to cover the near-24 h circadian cycle, with increasing session frequency around the theoretical evening wake- and morning sleep-maintenance zones, i.e. the times at which the circadian system maximally promotes alertness and sleep, respectively (clock times for 12PM-8AM habitual sleep schedule: 11AM, 5PM, 9PM, 11PM, 2AM, 6AM, 8AM, 11AM) (Fig. 1A). All TMS/EEG recordings were carried out while participants perform a visuo-motor vigilance task to exclude vigilance lapses from analyses^30^. This task consisted of maintaining a constantly moving red cursor in the center of a black screen (center indicated by a white dot) using a very sensitive tracking ball (light level remained <5 lux at all times), which required very limited movement of a single finger of the dominant hand. A lapse was defined as a time when the cursor was located outside of a central 200 by 200 pixel box surrounding target following >500 msec from the last trackball movement. The lapse period included the period between the last trackball movement and the lapse detection. TMS evoked responses occurring during and <1 s from a lapse were discarded from the analyses. Saliva samples were collected hourly throughout the protocol for melatonin assays, which were used as proxies for amplitude and phase of the circadian timing system^28^. Subjective sleepiness was assessed every hour throughout the protocol, including after each TMS-EEG assessment.

### Data acquisition

TMS/EEG data were recorded with a 60-channel TMS-compatible EEG amplifier (Eximia EEG, Nexstim, Helsinki, Finland), and TMS were delivered by means of a Focal Bipulse 8-Coil (Eximia TMS) combined with a magnetic resonance–guided navigation system (Eximia NBS). TMS pulses were delivered every 2000 ms (jitter 1900–2200 ms). The EEG amplifier gates the TMS artefact and prevents saturation by means of a proprietary sample-and-hold circuit that keeps the analog output of the amplifier constant between 100 μs before and 2 ms after the stimulus^45^. This guarantees the absence of TMS-induced magnetic artefacts from 8 ms post-TMS. Importantly, Eximia TMS and NBS are validate for pre-surgical use (FDA approval) guaranteeing the precision of TMS pulse delivery. At the end of each experiment, electrode positions on the participant’s head were recorded via the neuronavigation system. Two extra electrodes were used for electrooculograms. During EEG recordings, participant’s perception of the clicks produced by TMS coil discharge were eliminated using earplugs continuously playing a white masking noise (<90 dB, adjusted prior to each recording)^30^. Bone conductance was minimized by applying a thin foam layer between EEG cap and TMS coil^32^. The absence of auditory perception as verified through a short sham session performed at the end of each TMS-EEG recording during which 40 TMS pulse were delivered parallel to the scalp such that noise production was equal but TMS was not eliciting neuronal responses. In all cases, no auditory evoked response was detected in any of the session and in any of the subjects. All electrodes impedances were below 5 kΩ. Signals were band-pass filtered between 0.1–500 Hz and sampled at 1450 Hz. During TMS/EEG pretest, TMS location, orientation and intensity were adjusted for each participant to obtain a TMS/EEG evoked response with first component amplitude between 5–10 μV from peak-to-peak at the closest electrode to the TMS coil.

### Data analyses

TMS-hdEEG data were preprocessed and analyzed with Statistical Parametric Mapping 12 (SPM12, http://www.fil.ion.ucl.ac.uk/spm/), and analyzed with MATLAB^®^ (2011a, The Mathworks Inc, Natick, MA). Data were visually inspected for artifacted channels and trials. Trials corresponding to vigilance lapses based on the simultaneous vigilance tasks were excluded. EEG signals were re-referenced to the average of all good channels. Continuous EEG recordings were low-pass filtered at 80 Hz, downsampled from 1450 to 1000 Hz and high-pass filtered at 1 Hz. They were split into epochs −100 and 300 ms pre and post TMS pulses. Baseline correction was computed using −100 to −1.5 ms pre TMS pulse data. Robust averaging was applied to compute the mean evoked response of each session^46^.

### Frequency analyses of TMS-evoked responses

Frequency domain responses were calculated for averaged oscillatory TMS/EEG evoked activity, based on Morlet wavelets in SPM12. Frequency activity for the beta (20–29 Hz) and gamma range (30–50 Hz) were computed for the electrode nearest to the TMS stimulation at the individual level, during the time window of 5–50 ms.

### Dynamic Causal Modelling applied to TMS-evoked responses

Dynamic Causal Modeling (DCM) is a physiologically-driven approach to investigate how brain connectivity is affected by context, based on a realistic generative model^33^. DCM uses Neural Mass Models (NMM) to generate responses in laminar-specific populations of excitatory/inhibitory cells (Fig. 3A)^36^,^37^. NMM includes physiologically meaningful neuronal “characteristics” (membrane potentials, ionic-conductance, pre- and post-synaptic responses, and so forth), and summarizes neuronal states within a cortical macrocolumn and describes their interactions. Here, NMM assumes that a given cortical area comprises 4 subpopulations of neurons (deep and superficial pyramidal cells, excitatory stellate cells and inhibitory interneurons). These subpopulations have excitatory and inhibitory projections among each other and exhibit self-inhibition controlling neuronal gain. NMM also includes 3 of the time constant of NMDA, AMPA and GABAa receptors, voltage-gated ion channels (K^+^, Na^+^, Cl^−^, Ca^2+^), axonal delays from superficial to deep layers, and mean post-synaptic firing rate^36^. DCM for evoked responses allows for inference on how different NMM parameters contribute to the observed evoked response. We assumed that the TMS pulse represented the input as would be typically induced by sensory stimuli – and formed a Gaussian bump function input to the middle layer IV cell ensemble of spiny stellate cells. To estimate how internal NMM parameters vary in concert within a given experimental scenario, DCM uses generative or forward models for evoked EEG responses and fits these models using a variational Bayesian inversion scheme^33^. Here, we modeled the active source as the individual MRI coordinate of the TMS hotspot (cortical area maximally stimulated by TMS) within the SMA, by means of a single equivalent current dipole (ECD) within an electromagnetic forward model over the 5–50 ms post-TMS window. During this period most changes in the EEG can arguably be attributed to the sole TMS hotspot. This model used a “boundary element method” approach, with homogeneously and isotropically conductive volumes delimited by the brain, cerebrospinal fluid (CSF), outer skull and scalp surfaces. Individual head models are derived using an inverse spatial normalization of a canonical mesh for each participant (MRI T1-sequence, 20400 dipoles). Coregistration of the 60 electrode positions and head model was performed in each participant before forward model computation. A lead-field mapping of cortical sources onto measured signals was parameterized for orientation and location of the ECD^36^.

Here we used a single fully-free parameter DCM, whereby all 20 parameters of the NMM were allowed to vary in the experimental setup across TMS/EEG sessions. These parameters were chosen to allow for the model to recreate the multifaceted physiological and synaptic changes under sleep deprivation^47^.

### Neuronal Excitation/Inhibition balance based in Dynamic causal modeling

To test whether time-of-day impacts on excitatory/inhibitory drive, two proxies for these drives were used. First, neurotransmitter system parameters were identified, which do not account for the synchronicity but simply the shape of the EPSP/IPSP. This index was therefore a model-based metric of GABA/Glutamate receptor function balance^18^ based on the time constants of dynamic of conductances across these receptor-gated ion channels. Second, cell-to-cell connectivity parameters were used to calculate the difference between excitatory connections from stellate and deep pyramidal cells to inhibitory interneurons and the inhibitory connections from inhibitory interneurons to stellate and superficial pyramidal cells. This index thus considers connectivity between neuronal populations and accounts for the synchronicity between cell ensembles in the model^2^.

The indices were constructed as follows:

- GABA/Glutamate balance: GABAaR receptor time constants, where larger values mean that I/EPSPs have slower, more prolonged dynamics. *minus* sum of fast AMPAR and NMDAR receptor time constants, where larger values mean that I/EPSPs have faster, shorter dynamics.
- Neuronal cell-to-cell excitation/inhibition balance: sum of excitatory connections from stellate and deep pyramidal cells to inhibitory interneurons *minus* sum of inhibitory connections from inhibitory interneurons to stellate and superficial pyramidal cells.

### Cortical excitability

Detailed results of the analyses of cortical excitability changes with wakefulness extension and their relationships with other aspect of brain function and physiology and behavior have already been reported^17^. The amplitude and slope of the first EEG component (0–30 ms) of the TMS evoked potential (TEP) measured at the artifact free electrode closest from the hotspot (i.e. brain location with highest TMS-induced electrical field estimated by the neuronavigation system) were used as proxies for cortical excitability. The latter electrode was always located in the stimulated brain hemisphere. It could vary across participants but remained constant at the individual level. Results regarding cortical excitability are published elsewhere^17^ but were used for correlation analyses with DCM excitation/inhibition indices.

### Spontaneous quiet waking EEG

Data were analyzed with SPM12 implemented in MATLAB^®^ Channels and epochs with artefacts (eye blinks, body movements, slow eye movements) were rejected after visual inspection. Continuous EEG recordings were downsampled from 1450 to 500 Hz. Power spectral densities were computed using Welch’s methods (pwelch function in MATLAB 7.5), which consist in splitting the data segment into overlapping windows, processing these, then averaging the spectra across them. Power density of artifact-free 4-s epochs was averaged over the 2 min epoch. EEG activity was computed over frontal region (FP1, FPz, FP2, AF1, AFz, AF2, F7, F3, F1, Fz, F2, F4, F8) for delta (0.75–4 Hz), theta (4.5–7.5 Hz), alpha (8–12 Hz), sigma (12.5–18 Hz) and beta (18.5–30 Hz) frequency bands over the entire 2-min recording.

### Saliva collection and melatonin assays

Saliva samples were first placed at 4 °C, prior centrifugation and congelation at −20 °C within 12 h. Salivary melatonin was measured by radioimmunoassay (Stockgrand Ltd, Guildford, UK), as previously described^48^. Of a total of 624 samples, 546 were analyzed in duplicate. The limit of detection of the assay for melatonin was 0.8 ± 0.2 pg/ml using 500 μL volumes.

Estimation of circadian phase (where 0° = individual Dim Light Melatonin Onset - DLMO) was determined based on raw values. The first 4 samples were disregarded and maximum secretion level was set as the median of the 3 highest concentrations during the constant routine. Baseline level was set to be the median of the values collected from wake-up time +5 h to wake-up time +10 h. DLMOn was computed as time at which melatonin level reach 20% of the baseline to maximum difference (following linear interpolation).

### Statistics

All statistical analyses were performed with SAS version 9.3 (SAS Institute, Cary, NC, USA). All data were realigned according to circadian phase determined from individual melatonin profiles. All DCM-parameters and TMS/EEG evoked responses were *z*-scored to provide normalized amplitudes. TMS vigilance task was normalized by dividing performance to the duration of task and then z-scored. Frontal waking EEG activity in the different frequency bands were normalized by dividing power in a given band by the sum of frequencies within 0.75 and 30 Hz over the same region. To examine the time-course of the DCM excitation/inhibition drive, comparisons were made with mixed-model analyses of variance for repeated measures (PROC Mixed), with within-subject factor “time”. Contrasts were assessed with the LSMEANS statement. All *p*-values were based on Kenward-Roger’s corrected degrees of freedom. Post hoc p-values sought for significant least square mean different, i.e. time course values significantly different from the mean, and were corrected for multiple post hoc using Tukey-Kramer correction. Estimation of circadian phase (where 0° = individual DLMO) was determined based on raw values. The 4 first samples were disregarded and maximum secretion level was set as the median of the 3 highest concentrations during the constant routine. Baseline level was set to be the median of the values collected from wake-up time +5 h to wake-up time +10 h. Dim Light Melatonin Onset (DLMOn) was computed as time at which melatonin level reach 20% of the baseline to maximum difference (following linear interpolation).

To estimate how excitatory-inhibitory parameters (neuronal cell-to-cell connectivity and GABA/Glutamate balance) were associated to TMS-evoked EEG response (cortical excitability, beta and gamma frequency band power), spontaneous waking EEG power bands, subjective sleepiness and vigilance task behavioral responses, Spearman correlation analyses were performed to estimate the relationship across these variables. Furthermore, Analyses of Covariance (PROC GLM, SAS) were performed to estimate the relationship between neuronal cell-to-cell connectivity and GABA/Glutamate balance and these macroscopic system-level changes across circadian phases.

## Additional Information

**How to cite this article**: Chellappa, S. L. *et al*. Circadian dynamics in measures of cortical excitation and inhibition balance. *Sci. Rep.* **6**, 33661; doi: 10.1038/srep33661 (2016).

## Supplementary Material

Supplementary Information

## Figures and Tables

**Table 1:** Demographic characteristics (n = 22; Mean ± Standard deviation), together with sleep-wake timings from sleep diary and actigraphy data (Median ± Standard deviation).

N	22
AGE	22.82 ± 2.61
ETHNICITY	Caucasians
BODY MASS INDEX	22.23 ± 2.05
ANXIETY LEVEL (BDII)	1.23 ± 1.93
MOOD (BECK)	1.68 ± 2.12
DAYTIME PROPENSITY TO FALL ASLEEP (ESS)	3.73 ± 2.73
CHRONOTYPE (HO)	52.41 ± 5.03
RIGHT HANDED	17/22
SLEEP QUALITY (PSQ)	4.09 ± 0.15
SEASONALITY (SPAQ)	0.64 ± 0.79
CAFFELINE (cup/day)	0.41 ± 0.50
ALCOHOL (unit/week)	3.41 ± 0.20
CHRONOTYPE (MCTQ)	4.76 ± 0.16
SLEEP TIME (Sleep diary)	23:25 ± 0:20
WALK TIME (Sleep diary)	7:30 ± 0:17
SLEEP DURATION (Sleep diary)	8:10 ± 0:15
SLEEP TIME (Actigraphy)	23:30 ± 0:15
WALK TIME (Actigraphy)	7:30 ± 0:20
SLEEP DURATION (Actigraphy)	8:00 ± 0:20

ANXIETY LEVEL was measured on the 21 item Beck Anxiety Inventory^49^ (BAI≤19); CHRONOTYPE was assessed by the Horne‐Ösberg Questionnaire^50^ (31<HO<69); Daytime propensity to fall asleep in non‐stimulating situations was assessed by the Epworth Sleepiness Scale^51^ (ESS≤10); MOOD was assessed using the 21-item Beck Depression Inventory II^52^ (BDI‐II≤19); SLEEP QUALITY was determined by the Pittsburgh Sleep Quality Index Questionnaire^53^ (PSQI≤5). SEASONALITY is based on the Seasonal Pattern Assessment Questionnaire^54^ (SPAQ). The Edinburgh Inventory^55^ was administered to verify that the participants were right‐handed. SLEEP DIARY and ACTIGRAPHY measures were collected during 7-days prior to the study (Data presented in hours).

**Table 2 t2:** Characteristics of the 8 h baseline night of sleep immediately preceding the sleep deprivation paradigm (n = 22; Mean ± Standard error of mean).

Baseline night Sleep Structure	
Total Sleep Time (h)	7.4 ± 0.15
Sleep Efficiency (%)	92.5 ± 1.9
Wake (min)	27 ± 4.3
NREM stage 1 (min)	63 ± 4.5
NREM stage 2 (min)	220 ± 7.9
NREM stage 3 (min)	78 ± 6.2
REM (min)	85 ± 4.1
