# Supplementary material for: Circadian dynamics in measures of cortical excitation and inhibition balance
Source: Sci Rep. 2016 Sep 21;6:33661. doi: 10.1038/srep33661 (PMC5030482; doi:10.1038/srep33661)
Supplement: Supplementary Information [file srep33661-s1.doc]

**Circadian dynamics in measures of cortical excitation and inhibition balance**

*Abbreviated title:* Circadian excitation/inhibition balance

Sarah L. Chellappa^a,b,1^, Giulia Gaggioni^a,b,1^, Julien Q. M. Ly^a,b,c,1^, Soterios Papachilleos^a,b^, Chloé Borsu^a,b^, Alexandre Brzozowski^a,b^, Mario Rosanova^d,e^, Simone Sarasso^d^, André Luxen^a,b^, Benita Middleton^f^, Simon N. Archer^f^, Derk-Jan Dijk^f^, Marcello Massimini^d^, Pierre Maquet^a,b,c^, Christophe Phillips^a,b^, Rosalyn J. Moran^g,2^, Gilles Vandewalle^a,b,2,3^

**ONLINE SUPPLEMENTARY INFORMATION**

^a^ GIGA-Research, Cyclotron Research Center-In Vivo Imaging Unit, 8 allée du Six Août, Batiment B30, University of Liège, 4000 Liège, Belgium

^b^ Walloon excellence in life sciences and biotechnology (WELBIO, Belgium)

^c^ Department of Neurology, Domaine Universitaire du Sart Tilman, Bâtiment B35, CHU de Liège, 4000 Liège, Belgium

^d^ Department of Biomedical and Clinical Sciences “E.Sacco”, Università degli Studi di Milano, via G. B. Grassi 74, 20157 Milano, Italy

^e^ Fondazione Europea di Ricerca Biomedica, Ferb Onlus, Milan, Italy

^f^ Surrey Sleep Research Centre, Faculty of Health and Medical Sciences, University of Surrey, GU2 7XP Guildford, United Kingdom

^g^ Virginia Tech Carilion Research Institute & Bradley Department of Electrical and Computer Engineering, Virginia Tech, 2 Riverside Circle, VA 24016 Roanoke, USA

*^1^ These authors equally contributed to this work*

*^2^ Shared senior authorship*

**
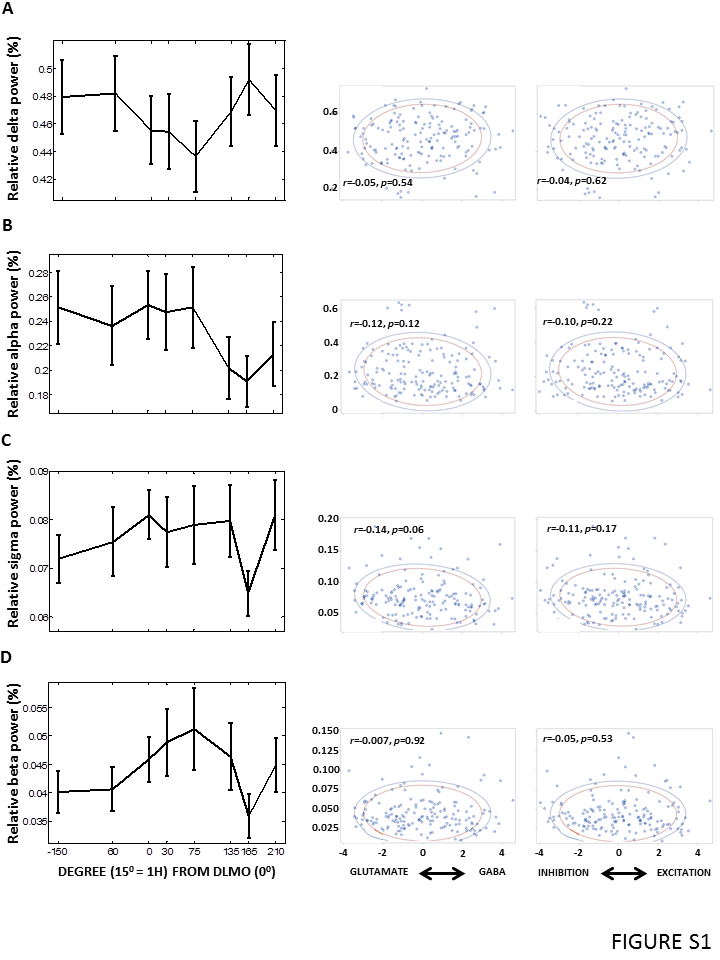
**

**Figure S1. Changes in frontal EEG power across 29h of sustained wakefulness were not correlated with DCM indices of excitation/inhibition balance.**

On all plots n = 22, and data are realigned according to individual melatonin secretion onset (phase 0°). Horizontal axis corresponds to time in degrees (15° = 1h) relative to melatonin secretion onset.

**A.** Relative delta power (0.75-4Hz) did not change significantly across the protocol (main effect of circadian phase: F_7,109_= 1.53, p = 0.17) and was not correlated to GABA/Glutamate receptor density balance (Spearman correlations; *r* = 0.05, *p =* 0.54; middle panel) and cell-to-cell excitation/inhibition connectivity balance (Spearman correlations; *r* = 0.04, *p =* 0.62; right panel).

**B.** Relative alpha power (8-12Hz) changed significantly across the protocol (main effect of circadian phase: **F_7,109_= 3.79, p = 0.001**) but was not correlated to GABA/Glutamate receptor density balance (Spearman correlations; *r* = -0.12, *p =* 0.12; middle panel) and cell-to-cell excitation/inhibition connectivity balance (Spearman correlations; *r* = -0.1, *p <* 0.22; right panel).

**C.** Relative sigma power (12.5-18Hz) did not change significantly across the protocol (main effect of circadian phase: F_7,111_= 1.73, p = 0.11) and was not correlated to GABA/Glutamate receptor density balance (Spearman correlations; *r* = -0.14, *p =* 0.06; middle panel) and cell-to-cell excitation/inhibition connectivity balance (Spearman correlations; *r* = -0.11, *p =* 0.17; right panel).

**D.** Relative beta power (18.5-30Hz) did not change significantly across the protocol (main effect of circadian phase: F_7,112_= 1.84, p = 0.09) and was not correlated to GABA/Glutamate receptor density balance (Spearman correlations; *r* = -0.007, *p =* 0.92; middle panel) and cell-to-cell excitation/inhibition connectivity balance (Spearman correlations; *r* = -0.05, *p =* 0.53; right panel).


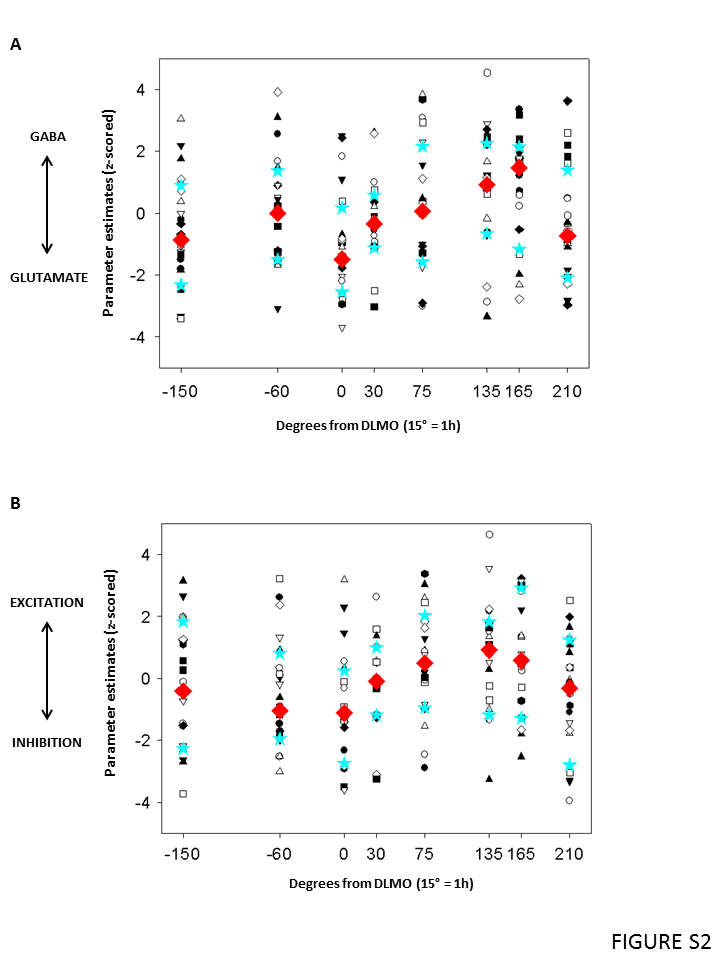
 **Figure S2. Individual changes in GABA/Glutamate receptor time constant and cell-to-cell excitation/inhibition connectivity balances during normal waking and sleep deprivation (related to figure 3).**

On both plots n = 22, and data are realigned according to individual melatonin secretion onset (phase 0°). Horizontal axis corresponds to time in degrees (15° = 1h) relative to melatonin secretion onset.

**Red diamond**s: median values; **Blue starss**: 20% and 80% percentiles

**A.** GABA/Glutamate receptor time constant balance variations (z-scored parameters at the individual level, as on figure 3).

**B.** Excitation/inhibition cell-to-cell connectivity parameter balance variations (z-scored parameters at the individual level, as on figure 3)

Overall both plots indicate that there is inter-individual variability in the variations of both indices. Therefore, in addition to the significant changes reported in the main text which we relate to changes in sleep homeostasis and circadian phase, there are other sources of variations in the indices of excitation and inhibition we computed. These interindividual variations should be investigated in future experiments.
